# Antiproliferation and cell apoptosis inducing bioactivities of constituents from *Dysosma versipellis *in PC3 and Bcap-37 cell lines

**DOI:** 10.1186/1747-1028-6-14

**Published:** 2011-06-15

**Authors:** Xiaoqiang Xu, Xiuhong Gao, Linhong Jin, Pinaki S Bhadury, Kai Yuan, Deyu Hu, Baoan Song, Song Yang

**Affiliations:** 1 State Key Laboratory Breeding Base of Green Pesticide and Agricultural Bioengineering, Key Laboratory of Green Pesticide and Agricultural Bioengineering, Ministry of Education, Guizhou University, Guiyang 550025, China

## Abstract

**Background:**

Recently, interest in phytochemicals from traditional Chinese medicinal herbs with the capability to inhibit cancer cells growth and proliferation has been growing rapidly due to their nontoxic nature. *Dysosma versipellis *as Bereridaceae plants is an endemic species in China, which has been proved to be an important Chinese herbal medicine because of its biological activity. However, systematic and comprehensive studies on the phytochemicals from *Dysosma versipellis *and their bioactivity are limited.

**Results:**

Fifteen compounds were isolated and characterized from the roots of *Dysosma versipellis*, among which six compounds were isolated from this plant for the first time. The inhibitory activities of these compounds were investigated on tumor cells PC3, Bcap-37 and BGC-823 *in vitro *by MTT method, and the results showed that podophyllotoxone (PTO) and 4'-demethyldeoxypodophyllotoxin (DDPT) had potent inhibitory activities against the growth of human carcinoma cell lines. Subsequent fluorescence staining and flow cytometry analysis indicated that these two compounds could induce apoptosis in PC3 and Bcap-37 cells, and the apoptosis ratios reached the peak (12.0% and 14.1%) after 72 h of treatment at 20 *μ*M, respectively.

**Conclusions:**

This study suggests that most of the compounds from the roots of *D. versipellis *could inhibit the growth of human carcinoma cells. In addition, PTO and DDPT could induce apoptosis of tumor cells.

## Background

Cancer is the major cause of human deaths worldwide because of its high incidence and mortality. Thousands of people die of cancer each year despite aggressive treatment regimens that include surgery, chemotherapy and radiotherapy. Due to the infiltrative nature and the rapid recurrence of the malignant tumor, complete surgical resection of these tumors is typically not achieved [1,2], and the conventional radiation and chemotherapy are often intolerable due to the strong systemic toxicity and local irritation [3-5]. These factors highlight the urgent need for new therapies or therapeutic combinations to improve the survival and quality of life of cancer patients.

In recent years, interest in phytochemicals from traditional Chinese medicinal herbs has been growing rapidly due to their ability to inhibit the growth and proliferation of cancer cells [6-9]. Due to their nontoxic nature, they are often employed in medical applications. Among of them, flavonoids and lignans present in traditional Chinese medicines have been revealed having significant activities against some forms of cancer [1,10]. The lignan podophyllotoxin is a plant toxin that exerts its cytotoxicity effect by inhibiting microtubule assembly and promoting cells to die via apoptosis [11]. However, it is not used as a clinical therapeutic agent due to its serious side effects. Extensive structure modifications were performed to obtain more potent and less toxic antitumor agents, e.g. etoposide [12] and teniposide [13] are currently used in anticancer therapy. Besides, some researchers found that podophyllotoxin and its derivatives had inhibitory effect on PC3 and other cells [14-16]. One of the popular Chinese herbal medicines, *Dysosma versipellis *(Hance) M. Cheng which belongs to Berberidaceae in *Dysosma *Family and is used as a source of podophyllotoxin [16] is a perennial herbaceous species grown in the understory of mixed evergreen and deciduous forests of China. In some folk remedies, *D. versipellis *(Hance) M. Cheng has been widely used to clear sputum, kill parasites and treat epidemic encephalitis B and epidemic parotitis. Previous studies have indicated that constituents and extracts of this traditional Chinese medicinal plant have growth inhibitory activities against various tumors *in vivo *or *in vitro *[16-18]. However, systematic and comprehensive studies have not been performed with regard to the chemical constituents from *D. Versipellis *and apoptosis-inducing effect of these components.

*Dysosma versipellis*, an endangered and endemic Bereridaceae plant species of China, has been proved to be an important Chinese herbal medicine because of its biological activity. In this study, fifteen compounds were isolated from the rhizomes of *D. versipellis *which was grown in Guizhou and identified by spectroscopic analysis and physicochemical data as *β*-sitosterol (**1**), 4'-demethylpodophyllotoxin (**2**), kaempferol (**3**), picropodophyllotoxin-4-*O-*glucoside (**4**), cleistanthin-B (**5**), kaempferol-3-*O-β-D*-glucopyranoside (**6**), 4'-demethylpodophyllotoxin-4-*O*-glucoside (**7**), quercetin3-*O-β- D*-glucopyranoside (**8**), icropodophyllotoxin-4-*O-β-D*-glucopyranosyl-(1→6)-*β-D*-glucopyranoside (**9**), quercetin (**10**), daucosterol (**11**), podophyllotoxone (PTO, **12**), vanillic acid (**13**), 4'-demethyldeoxypodophyllotoxin (DDPT, **14**), and sucrose (**15**). Among them, (**5**), (**7**), (**8**), (**12**), (**13**) and (**15**) were obtained from the plants for the first time. All the compounds were then bioassayed on human prostatic carcinoma cell line PC3, human breast cancer cell line Bcap-37, human gastric carcinoma cell line BGC-823 and mouse embryonic fibroblast cell line NIH3T3 *in vitro *by MTT method. It was found that DDPT had the most potent inhibitory activities against the growth of human carcinoma cell lines than other compounds extracted from *D. versipellis*. And PTO obtained from the plants for the first time also had high antitumor activity. There are a few reports on the anticancer effects of PTO and DDPT on various tumor cells recently [19-21]. And it was found that the inhibitory rate of PTO (100 *μ*g/mL) on P388 murine leukemia cell proliferation was 99.0%. Also PTO could induce HL-60 human leukemia cells apoptosis, which might be related with down-regulation of Bcl-2 expression. Other studies have found that DDPT possessed antitumor activity on A549 human lung carcinoma cell and SK-MEL-2 human melanoma cell with EC_50 _of 0.023 *μ*g/mL and 0.015 *μ*g/mL respectively. However, no report was found on the anticancer activities of PTO and DDPT on PC3, Bcap-37 and BGC-823 cells. This prompted us to study the anticancer activities of PTO and DDPT separated from the rhizomes of *D. versipellis *grown in Guizhou on the three kinds of cells mentioned above and investigate their preliminarily mechanism of action as potent anticancer agents. Thus, further investigations of PTO and DDPT on the three cells lines were carried out on PC3, Bcap-37, and BGC-823 cells. The IC_50 _of PTO and DDPT on the three cell lines were determined. Furthermore, experimental results of fluorescent staining and flow cytometry analysis indicated that PTO and DDPT could induce apoptosis in PC3 and Bcap-37 cells, with the apoptosis ratios of PC3 cells were 12.0% and 14.1% after 72 h of treatment at 20 *μ*M, respectively. To the best of our knowledge, this is the first report on apoptosis inducing and antitumor activity of PTO and DDPT on PC3, Bcap-37, and BGC-823 cells.

## Methods

### Analysis and Instruments

^1^H NMR, ^13^C NMR, and DEPT spectra were measured on a JOEL-ECX 500 MHz NMR spectrometer in CDCl_3_, CD_3_OD, or DMSO-*d*_6 _using tetramethylsilane (TMS) as an internal standard. The IR spectra were obtained in the KBr pellet using a SHIMADZU-IR Prestige-21 spectrometer. Melting points were determined on an XT-4 digital microscope (Beijing Tech Instrument Co.) Analytical TLC was performed on silica gel GF254 (400 mesh), and column chromatographic operations were performed on Silica gel (100-200 or 200-300 mesh, Qingdao Haiyang Chemical Co.). In addition, Sephadex LH-20 column chromatographic instrument (Beijing Huideyi Tech Instrument Co.) was employed for the extraction and purification of chemical composition.

### Plant material

The rootstalk of *D. versipellis *was collected in Qingzhen, Guizhou Province, China, in the month of May 2008. The voucher specimen was identified as *D. versipellis *(hance.) M.Cheng by Qing-De Long, the Dean of teaching-research section, School of Pharmacy of Guiyang Medical University, and was submitted to our laboratory for further investigation.

### Extraction and isolation

The dried roots of *D. versipellis *(20 kg) from Guizhou Province were powdered and extracted by maceration in 80% industrial alcohol (50 L, each 4d) for three times. The extracts which filtered and washed with alcohol each time were combined and reduced in vacuo to afford 4 kg crude extract. A part of crude extract (950 g) was further extracted with petroleum ether, chloroform and ethyl acetate to obtain three fractions of 40 g, 2 kg and 280 g respectively.

The petroleum ether extract (40 g) was chromatographed on silica gel column (200-300 mesh) and eluted with a PE-EtOAc (20:1~0:1) gradient, and recrystallized with methanol to give compound **1 **(300 mg).

The chloroform extract (100 g) chromatographed on silica gel column (100-200 mesh) was eluted with CHCl_3_-MeOH (1:0~0:1) and PE-EtOAc (20:1~0:1) to obtain fractions 1-5, respectively. Compounds **2 **(100 mg) and **3 **(200 mg) was obtained by PTLC and eluting with CHCl_3_-MeOH (6:1 and 5:1) from fractions 1 and 2, respectively. The fraction 3 was evaluated by Sephadex LH-20 and eluted with acetone to afford compounds **4 **(15 mg) and **5 **(10 mg). And fraction 4 was eluted with PE-EtOAc (10:1) and recrystallized with acetone to give compound **12 **(20 mg). The filtrate from fraction 5 was eluted with PE-EtOAc (9:1 and 10:1, respectively) to afford compounds **13 **(25 mg) and **14 **(23 mg).

The ethyl acetate extract (55 g) was chromatographed on silica gel column (100-200 mesh) and eluted with CHCl_3_-MeOH (15:1~0:1) to obtain fractions 1-3. The fraction 1 was evaluated by Sephadex LH-20 (methanol) to give compound **10 **(50 mg). Compounds **6 **(140 mg) and **7 **(209 mg) were obtained by silica gel column chromatography (200-300 mesh) and eluting with CHCl_3_-MeOH (8:1) from fraction 2. The fraction 3 was further chromatographed on silica gel column (100-200 mesh) and eluted with CHCl_3_-MeOH (10:1~0:1) to give subfractions 1-3. Compound **8 **(30 mg) were obtained by Sephadex LH-20 (methanol) from subfraction 1. The subfraction 2 was recrystallized with CHCl_3_-MeOH (8:1) to afford compound **15 **(15 mg). The subfraction 3 was recrystallized with MeOH and CHCl_3_-MeOH (9:1) to give compound **9 **(19 mg) and compound **11 **(18 mg), respectively.

### Anticancer activity bioassay

#### Cell culture

Human prostate cancer cell line PC3, breast cancer cell line Bcap-37, and gastric cancer cell line BGC-823 were purchased from Institute of Biochemistry and Cell Biology, China Academy of Science and cultured in RPMI 1640 medium supplemented with 10% heat-inactivated fetal bovine serum (FBS). Mouse embryonic fibroblast cell line NIH 3T3 was also obtained from the same place and cultured in DMEM supplemented with 10% FBS. All cell lines were maintained at 37°C in a humidified 5% carbon dioxide and 95% air incubator.

#### MTT asssy

All tested compounds were dissolved in DMSO (1-100 *μ*M solution) and subsequently diluted in the culture medium before treatment of the cultured cells. Tested cells were plated in 96-well plates at a density 2×10^3 ^cells/well/100 *μ*L of the proper culture medium and treated with the compounds at 1 to 100 *μ*M for 72 h. In parallel, the cells treated with 0.1% DMSO served as control. An MTT assay (Roche Molecular Biochemicals, 1465-007) was performed 30 h later according to the instructions provided by Roche. This assay was based on the cellular cleavage of MTT into formazane which is soluble in cell culture medium. Any absorbance caused by formazan was measured at 595 nm with a microplate reader (BIO-RAD, model 680), which is directly proportional to the number of living cells in culture. The experiment was performed in triplicate. The percentage cytotoxicity was calculated using the formula.

#### AO/EB staining

Cells were seeded at a concentration of 5×10^4 ^cell/ml in a volume of 0.6 mL on sterile cover slip in 6-well tissue culture plates. Following incubation, the medium was removed and replaced with fresh medium plus 10% FBS and supplemented with podophyllotoxone and 4'-demethyldeoxypodophyllotoxin (20 *μ*M). After the treatment period, the cover slip with monolayer cells was inverted on the glass slide with 20 *μ*L of AO/EB stain (100 *μ*g/mL). The fluorescence was read on an IX71SIF-3 fluorescence microscope (OLYPUS Co., Japan).

#### Hoechst 33258 staining

Cells grown on sterile cover slip in 6-well tissue culture plates were treated with podophyllotoxone and 4'-demethyldeoxypodophyllotoxin (20 *μ*M) for a certain range of treatment time. The culture medium containing compounds was removed and the cells were fixed in 4% paraformaldehyde for 10 min. After washing twice with PBS, cells were stained with 0.5 mL of Hoechst 33258 staining (Beyotime) for 5 min. After washing twice with PBS, stained nuclei were observed under an IX71SIF-3 fluorescence microscope by using 350 nm excitation and 460 nm emission.

#### TUNEL assays

TdT-UTP nick end labeling (TUNEL) assays were performed with colorimetric TUNEL apoptosis assay kit according to the manufacturer's instructions (Beyotime). Cells grown in 6 well culture clusters were treated as mentioned in mitochondrial depolarization assay. In short, Bcap-37 cells grown in 6-well tissue culture plates were washed with PBS and fixed in 4% paraformaldehyde for 40 min. After washing once with PBS, cells were permeabilized with immunol staining wash buffer (Beyotime) for 2 min on ice. Cells were washed once with PBS again and incubated in 0.3% H_2_O_2 _in methanol at room temperature for 20 min to inactivate the endogenous peroxidases after which the cells were washed for three times with PBS. Thereafter, the cells were incubated with 2 *μ*L of TdT-enzymea and 48 *μ*L of Biotin-dUTP per specimen for 60 min at 37°C. After termination for 10 min, cells were incubated with streptavidin-HRP (50 *μ*L per specimen) conjugate diluted at 1:50 in diluent of streptavidin-HRP for 30 min. After washing three times with PBS, cells were incubated with diaminobenzidine (DAB) solution (200 *μ*L per specimen) for 10 min. This was again followed by washing with PBS for two times and the result was imaged under an XDS-1B inverted biological microscope (Chongqing photoelectric device CO.).

#### Flow cytometry analysis

Prepared PC3 cells (1×10^6^/mL) were washed twice with cold PBS and then re-suspended gently in 500 *μ*L binding buffer. Thereafter, cells were stained in 5 *μ*L Annexin V-FITC and shaked well. Finally, 5 *μ*L PI was added to these cells and incubated for 20 min in a dark place, analyzed by FACS Calibur, Becton Dickinson.

#### Statistical analysis

All statistical analyses were performed with SPSS 10.0. Data were analyzed by one-way analysis of variance (ANOVA). Mean separations were performed using the least significant difference method (LSD test). Each experiment had three replicates and all experiments were run three times with similar results. Measurements from all the replicates were combined and treatment effects analyzed.

## Results and Discussion

### Chemistry

**Figure 1:**
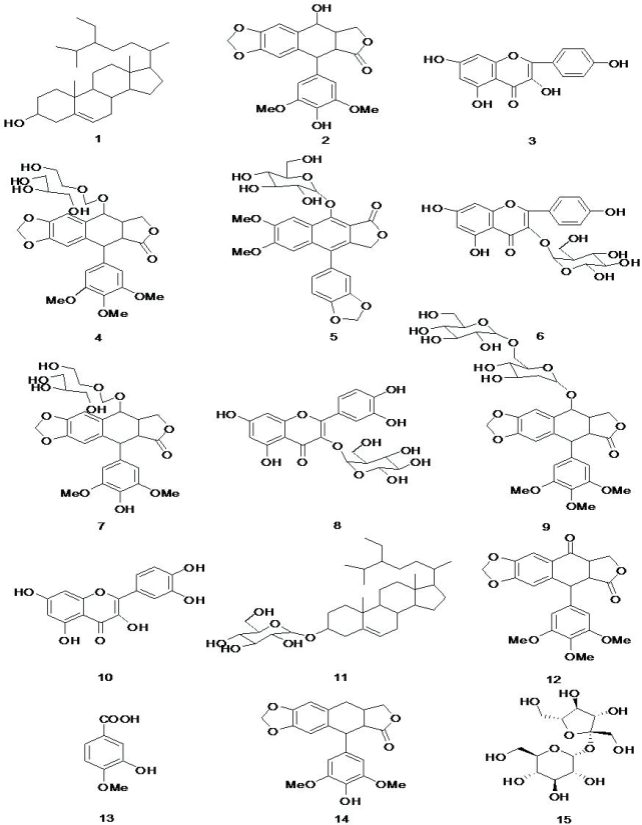
**The structures of compounds 1-15**. These compounds were obtained from the root of *Dysosma versipellis *and identified by spectroscopic analysis and physicochemical data.

The root of *D. versipellis *collected from Guizhou province was studied and fifteen compounds were isolated from the ethanol extracts, which were identified based on their physicochemical as well as spectroscopic data as *β*-sitosterol (**1**), 4'-demethylpodophyllotoxin (**2**), kaempferol (**3**), picropodophyllotoxin-4-*O-*glucoside (**4**), cleistanthin-B (**5**), kaempferol-3-*O-β-D*-glucopyranoside (**6**), 4'-demethylpodo-phyllotoxin-4-*O*-glucoside (**7**), quercetin-3-*O-β-D*-glucopyranoside (**8**), icropodo- phyllotoxin-4-*O-β-D*-glucopyranosyl-(1→6)-*β-D*-glucopyranoside (**9**), quercetin (**10**), daucosterol (**11**), podophyllotoxone (**12**), vanillic acid (**13**), 4'-demethyldeoxypodo-phyllotoxin (**14**), sucrose (**15**), as shown in Figure 1. It can be seen that these compounds can be classified as two steroids (compounds **1 **and **11**), four flavonoids (compounds **3**, **6**, **8 **and **10**), seven lignans (compounds **2**, **4**, **5**, **7**, **9**, **12 **and **14**), an organic acid (compound **13**), and sucrose (**15**).

Compound 1, white acerate crystal; m.p. 136~137°C; molecular formula: C_29_H_50_O; ^1^H NMR(CDCl_3_, 500 MHz) *δ*: 5.35 (1H, d, *J *= 2.6 Hz, H-6), 3.50~3.46 (1H, m, H-3), 0.68~2.29 (continuous peaks); ^13^C NMR(CDCl_3_, 125 MHz) *δ*: 140.8 (C-5), 121.8 (C-6), 71.9 (C-3), 56.8 (C-14), 56.1 (C-17), 50.2 (C-9), 45.9 (C-4), 42.4 (C-13), 39.9 (C-12), 37.3 (C-1), 36.5 (C-10), 36.2 (C-20), 32.0 (C-22), 34.0 (C-7), 32.0 (C-8), 31.8 (C-2), 29.2 (C-24), 28.3 (C-25), 28.2 (C-16), 26.2 (C-28), 24.4 (C-15), 23.2 (C-27), 21.2 (C-11), l9.9 (C-26), l9.5 (C-19), 19.1 (C-23), 18.9 (C-21), 12.1 (C-29), 11.9 (C-18). As analyzed above, it was identified as *β*-sitosterol [22].

Compound 2, colorless crystal; m.p. 234~237°C; molecular formula: C_21_H_20_O_8_; ^1^H NMR (CD_3_OD, 500 MHz) *δ*: 7.18 (1H, s, H-5), 6.49 (1H, s, H-8), 6.43 (2H, s, H-2', H-6'), 5.97 ( 2H, dd, *J *= 0.9 Hz, *J *= 1.0 Hz, OCH_2_O), 4.78 (1H, d, *J *= 4.8 Hz, H-4), 4.52 ~4.40 (2H, m, H-1, H-3a*α*), 4.12 (1H, t, *J *= 9.6 Hz, H-3a*β*), 3.70 (6H, s, 3', 5' -OCH_3 _), 3.02 (1H, dd, *J *= 9.5 Hz, *J *= 2.5 Hz, H-2), 2.84 (1H, m, H-3); ^13^C NMR (CD_3_OD, 125 MHz) *δ*: 174.4 (C-2a), 147.3 (C-3'), 147.3 (C-5'), 147.0 (C-6 ), 147.0 (C-7), 135.2 (C-1'), 135.0 (C-4), 131.7 (C-9), 131.3 (C-10), 109.3 (C-8), 109.1 (C-6'), 109.1 (C-2'), 106.5 (C-5), 101.3 (OCH_2_O), 71.8 (C-3a), 71.0 (C-4), 55.9 (3', 5'-OMe), 44.8 (C-2), 44.0 (C-1), 40.7 (C-3). As analyzed above, it was identified as 4'-demethyl- podophyllotoxin [23].

Compound 3, yellow powder; m.p. 281~283°C; molecular formula: C_15_H_10_O_6_; ^1^H NMR (CD_3_OD, 500 MHz) *δ*: 12.05 (1H, s, 5-OH), 8.03 (2H, d, *J *= 9.2 Hz, H-2', H-6' ), 6.90 (2H, d, *J *= 9.2 Hz, H-3', 5'), 6.40 (1H, d, *J *= 1.8 Hz, H-8), 6.14 (1H, d, *J *= 2.3 Hz, H-6); ^13^C NMR (CD_3_OD, 125 MHz) *δ*: 175.8 (C-4), 164.1 (C-7), 161.3 (C-5), 159.3 (C-4'), 157.0 (C-9), 146.2 (C-2), 135.8 (C-3), 129.6 (C-6'), 129.6 (C-2'), 122.5 (C-1'), 115.5 (C-3'), 115.4 (C-5), 103.3 (C-10), 98.3 (C-6), 93.7 (C-8). As analyzed above, it was identified as kaempferol [24].

Compound 4, white acerate crystal; m.p. 228~230°C; molecular formula: C_28_H_32_O_13_; ^1^H NMR (DMSO-*d_6_*, 500 MHz) *δ*: 7.29 (1H, s, H-5), 6.58 (2H, s, H-2', H-6'), 6.09 (1H, s, H-8), 5.92 (2H, d, *J *= 5.2 Hz, OCH_2_O), 4.68 (1H, d, *J *= 8.6 Hz, H-4), 4.62 (1H, d, *J *= 8.6 Hz, H-3a*α*), 4.48 (1H, d, *J *= 4.0 Hz, H-3a*β*), 3.97 (1H, d, *J *= 6.9 Hz, H-1), 3.73 (6H, s, 3'-OMe, 5'-OMe), 3.66 (3H, s, 4'-OMe), 3.60 (1H, d, *J *= 3.2 Hz, H-2), 2.84~2.66 (1H, m, H-3), Glc: 5.06 (1H, d, *J *= 7.5 Hz, H-1''), 4.46 (1H, d, *J *= 4.6 Hz, H-3''), 4.40 (1H, dd, *J *= 6.8 Hz, *J *= 3.5 Hz, H-6''), 3.09~3.21 (4H, m, H-2'', H-4''~H-6''); ^13^C NMR (DMSO-*d_6_*, 125 MHz) *δ*:178.2 (C-2a), 153.3 (C-3', 5'), 146.7 (C-6), 146.2 (C-7), 138.7(C-1'), 136.5 (C-4'), 132.1 (C-9), 132.0 (C-10), 107.8 (C-5), 107.6 (C-8), 106.6 (C-2', 6'), 101.2 (OCH_2_O), 77.5 (C-4), 70.2 (C-11), 60.5 (4'-OCH_3 _), 56.3 (3', 5'-OCH_3 _), 43.9 (C-1), 44.1 (C-2), 41.9 (C-3), Glc: 104.1 (C-1''), 74.3 (C-2''), 77.3 (C-3'', 5''), 70.04 (C-4''), 61.6 (C-6''). As analyzed above, it was identified as picropodophyllotoxin-4-*O- *glucoside [25].

Compound 5, white powder; m.p. 154~156°C; molecular formula: C_27_H_26_O_12_; ^1^H NMR(CD_3_OCD_3_, 500 MHz) *δ*: 8.28 (1H, s, H-5), 7.11 (1H, d, *J *= 1.7 Hz, H-5'), 6.98 (1H, dd, *J *= 5.2 Hz, *J *= 2.3 Hz, H-8), 6.90 (1H, d, *J *= 1.8 Hz, H-2'), 6.88 (1H, dd, *J *= 7.5 Hz, *J *= 2.3 Hz, H-6'), 6.10 (2H, s, 3', 4'-OCH_2_O), 5.60 (2H, dd, *J *= 21.0 Hz, *J *= 2.3 Hz, H-3a), 4.94 (1H, d, *J *= 8 Hz, H-1''), 4.00 (3H, s, 6-OMe), 3.96 (1H, d, *J *= 6.3 Hz, H-6''), 3.74 (3H, s, 7-OMe), 3.69 (1H, d, *J *= 4 Hz, H-6''), 3.51~3.49 (4H, m, H-2''~H-5''); ^13^C NM R (CD_3_OCD_3_, 125 MHz) *δ*: 136.5 (C-1), 120.2 (C-2), 128.2 (C-3), 146.2 (C-4), 102.2 (C-5), 153.0 (C-6), 151.5 (C-7), 106.7 (C-8), 131.6 (C-9), 131.3 (C-10), 129.8 (C-1'), 111.7 (C-2'), 148.3 (C-3'), 148.4 (C-4'), 108.7 (C-5'), 124.6 (C-6'), 169.9 (C-2a), 68.1 (C-3a), 56.4 (6-OMe), 55.7 (7-OMe), 102.7 (OCH_2_O), 106.3 (C-1''), 73.4 (C-2''), 75.2 (C-3''), 71.4 (C-4''), 78.1 (C-5''), 62.8 (C-6''). As analyzed above, it was identified as cleistanthin-B [26].

Compound 6, yellow crystal; m.p. 196~198°C, molecular formula: C_21_H_20_O_11_; ^1^H NMR (CD_3_OD, 500 MHz) *δ*: 8.03 (2H, d, *J *= 8.6 Hz, H-2', 6'), 6.87 (2H, d, *J *= 8.6 Hz, H-3', 5'), 6.36(1H, d, *J *= 2.3 Hz, H-8), 6.17 (1H, d, *J *= 2.3 Hz, H-6), 5.23(1H, d, *J *= 7.4 Hz, H-1''); ^13^C NMR (CD_3_OD, 125 MHz) *δ*: 178.1 (C-4), 164.1 (C-7), 161.2 (C-5),160.2 (C-4'), 157.6 (C-2), 157.2 (C-9), 134.0 (C-3), 130.9 (C-2'), 130.9 (C-6'), 120.9 (C-1'), 114.7 (C-3'), 114.7 (C-5'), 104.2 (C-10), 98.7 (C-6), 93.5 (C-8), 102.7 (C-1''), 74.4 (C-2''), 77.0 (C-3''), 70.0 (C-4''), 76.7 (C-5''), 61.2 (C-6''). As analyzed above, it was identified as kaempferol-3-*O-β-D*-glucopyranoside [27].

Compound 7, white power; m.p. 140~142°C; molecular formula: C_27_H_30_O_13_; ^1^H NMR (CD_3_OD, 500 MHz) *δ*: 7.38 (1H, s, H-5), 6.40 (2H, s, H-2', H-6'), 5.94 (2H, dd, *J *= 1.2 Hz, *J *= 1.2 Hz, OCH_2_O), 5.05 (1H, d, *J *= 4.9 Hz, H-4), 4.69 (1H, dd, *J *= 7.7 Hz, *J *= 3.5 Hz, H-3a*α*), 4.54 (1H, d, *J *= 2.3 Hz, H-1), 4.39 (1H, d, *J *= 3.7 Hz, H-1''), 4.21 (1H, t, *J *= 9.5 Hz, H-3a*β*), 3.90 (1H, d, *J *= 7.5 Hz, H-6''), 3.88 (1H, d, *J *= 7.5 Hz, H-6''), 3.72 (6H, s, 3', 5'-OMe), 3.35 (4H, m, H-2''~ 5''), 3.03 (1H, dd, *J *= 9.7 Hz, *J *= 2.6 Hz, H-2), 2.95 (1H, m, H-3); ^13^C NMR (CD_3_OD, 125 MHz) *δ*: 175.7 (C-2a), 147.7 (C-3', 5'), 147.2 (C-6, 7), 134.4 (C-4'), 132.1 (C-1'), 131.2 (C-10), 131.1 (C-9), 108.8 (C-8), 108.3 (C-2', 6'), 108.2 (C-5), 102.3 (C-1''), 101.2 (OCH_2_O), 79.0 (C-5''), 76.8 (C-3''), 73.8 (C-2''), 71.5 (C-4), 70.3 (C-3a), 70.2 (C-4''), 61.4 (C-6''), 55.5 (3', 5'-OCH_3_), 45.1 (C-2), 43.7 (C-1), 39.2 (C-3). As analyzed above, it was identified as 4'-demethylpodophyllotoxin-4-*O*-glucoside [28].

Compound 8, yellow crystal; m.p. 188~190°C; molecular formula: C_21_H_20_O_12_; ^1^H NMR (CD_3_OD, 500 MHz) *δ*: 7.61 (1H, d, *J *= 1.8 Hz, H-2'), 6.77 (1H, d, *J *= 8.6 Hz, H-5'), 6.49 (2H, dd, *J *= 2.3 Hz, *J *= 6.3 Hz, H-6'), 6.29 (1H, d, *J *= 1.7 Hz, H-8), 6.10 (1H, d, *J *= 2.3 Hz, H-6), 5.16 (1H, d, *J *= 7.5 Hz, H-1''), 3.10~3.63 (6H, m, H-2''~6''); ^13^C NMR (CD_3_OD, 125 MHz) *δ*: 178.1 (C-4), 165.3(C-7), 161.7 (C-5),157.6 (C-2), 157.2 (C-9), 148.5 (C-4'), 144.6 (C-3' ), 134.2 (C-3), 121.8 (C-6'), 121.7 (C-1' ), 116.1 (C-5'), 114.6 (C-2' ), 104.2 (C-10), 102.9 (C-1''), 98.7 (C-6), 93.4 (C-8), 77.1 (C-3''), 76.8 (C-2''), 74.4 (C-4''), 69.8 (C-5''), 61.2 (C-6''). As analyzed above, it was identified as quercetin-3-*O-β-D*-glucopyranoside [29].

Compound 9, white crystal; m.p. 225~228°C; molecular formula: C_34_H_42_O_18_; ^1^H NMR (DMSO-*d_6_*, 500 MHz) *δ*: 7.21 (1H, s, H-5), 6.65 (2H, s, H-2', H-6'), 5.95 (2H, d, *J *= 6.3 Hz, OCH_2_O), 5.83 (1H, s, H-8), 5.36 (1H, d, *J *= 2.6 Hz, H-1), 5.14 (1H, d, *J *= 5.2 Hz, H-3a*β*), 4.71 (1H, d, *J *= 2.3 Hz, H-4), 4.07 (1H, d, *J *= 4.6 Hz, H-3a*α*), 3.76 (6H, s, 3', 5'-OMe), 3.68 (3H, s, 4'-OMe), 2.92 (1H, m, H-3), 3.20~3.18 (1H, m, H-2), Glc(inner): 5.13 (1H, d, *J *= 5.8 Hz, H-1'), 4.49 (1H, d, *J *= 7.5 Hz, H-2'), 3.90 (1H, t, *J *= 9.5 Hz, H-3'), 3.52~3.49 (1H, m, H-4'), 4.56~4.49 (1H, m, H-5'), 4.62 (1H, d, *J *= 10.3 Hz, H-6'), 4.44~4.33 (1H, m, H-6'); Glc (terminal): 4.81 (1H, d, *J *= 4.0 Hz, H-1''), 3.07 (1H, dd, *J *= 8.6 Hz, H-2''), 3.52~3.41 (1H, m, H-3''), 3.90 (1H, t, *J *= 9.5 Hz, H-4''), 3.42 (1H, m, H-5''), 4.57 (1H, d, *J *= 4.6 Hz, H-6''), 4.42~4.38 (1H, m, H-6''); ^13^C NMR (DMSO-*d_6_*, 125 MHz) *δ*: 178.4 (C-12), 153.4 (C-3',5'), 146.6 (C-6), 146.0 (C-7), 138.3 (C-1'), 136.4 (C-4'), 132.9 (C-10), 132.8 (C-9), 107.8 (C-8), 106.9 (C-2', 6'), 106.3 (C-5), 101.2 (OCH_2_O), 76.8 (C-4), 68.5 (C-11), 60.5 (4'-OCH_3 _), 56.4 (3', 5'-OCH_3_); Glc (inner): 104.1 (C-1'), 73.9 (C-2'), 77.4 (C-3', 5'), 70.8 (C-4'), 69.3 (C-6'); Glc (terminal): 103.6 (C-1''), 74.2 (C-2''), 77.3 (C-3'', 5''), 70.4 (C-4''), 61.4 (C-6''). As analyzed above, it was identified as icropodophyllotoxin-4-*O-β-D*-glucopyranosyl-(1→6)-*β-D*-glucopyranoside [23,30].

Compound 10, yellow power; m.p. 306~308°C; molecular formula: C_15_H_10_O_7_; ^1^H NMR (CD_3_OD, 500 MHz) *δ*: 7.64 (1H, d, *J *= 2.3 Hz, H-2'), 7.53 (1H, dd, *J *= 8.6 Hz, *J *= 2.3 Hz, H-6'), 6.78 (1H, d, *J *= 8.6 Hz, H-5'), 6.28 (1H, d, *J *= 2.3 Hz, H-8), 6.08 (1H, d, *J *= 1.8 Hz, H-6); ^13^C NMR (CD_3_OD, 125 MHz) *δ*: 175.9 (C-4), 164.3 (C-7), 161.2 (C-5), 156.9 (C-9), 147.4 (C-2), 146.6 (C-3'), 144.9 (C-4'), 135.9 (C-3), 122.8 (C-1'),120.3 (C-6'), 114.9 (C-5'), 114.6 (C-2'), 103.1 (C-10), 97.9 (C-6), 93.1 (C-8). As analyzed above, it was identified as quercetin [31,32].

Compound 11, white power; m.p. 297~300°C; molecular formula: C_35_H_60_O_6_; ^1^H NMR(Pyridine-*d*_5_, 500 MHz) *δ*: 5.35 (1H, s, H-6), 5.06~5.07 (1H, m, H-1'), 2.12~4.60 (10H, m, GluH), 0.85~1.13 (18H, m, H-CH_3_), 0.66 (3H, s, H-29); ^13^C NMR (Pyridine-*d*_5_, 125 MHz) *δ*: 139.0 (C-5), 120.2 (C-6), 100.8 (C-1'), 77.0 (C-5'), 76.9 (C-3'), 76.4 (C-3), 73.7 (C-2'), 70.0 (C-4'), 61.2 (C-6'), 55.2 (C-14), 55.1 (C-17), 54.6 (C-9), 54.4 (C-24), 49.7 (C-4), 48.7 (C-13), 44.4 (C-12), 40.8 (C-1), 39.1 (C-10), 38.3 (C-20), 38.1 (C-22), 37.7 (C-7), 35.8 (C-8), 35.3 (C-2), 34.7 (C-25), 32.5 (C-16), 30.5 (C-23), 30.4 (C-15), 28.7 (C-28), 27.8 (C-11), 27.6 (C-19), 26.9 (C-21), 24.7 (C-27), 24.0 (C-26), 22.8 (C-29), 16.0 (C-18). As analyzed above, it was identified as daucosterol [33].

Compound 12, colorless acerate crystal; m.p. 183~185°C; molecular formula: C_22_H_20_O_8_; ^1^H NMR(CD_3_COCD_3_, 500 MHz) *δ*: 7.43 (1H, s, H-5), 6.83 (1H, s, H-8), 6.49 (2H, s, H-2', 6'), 6.15 (2H, s, OCH_2_O), 4.91 (1H, d, *J *= 4.0 Hz, H-1), 4.49 (1H, t, *J *= 7.7 Hz, H- 3a*α *), 4.34 (1H, t, *J *= 9.5 Hz, H-3a*β*), 3.70 (6H, s, 3', 5'-OM e), 3.68 (3H, s, 4'-OMe), 3.65~3.49 (1H, m, H-3), 3.63 (1H, d, *J *= 3.5 Hz, H-2); ^13^C NMR (CD_3_COCD_3_, 125 Hz) *δ*: 192.2 (C-4 ), 173.1 (C-2a ), 153.1 (C-5'), 153.1 (C-7), 153.0 (C-3'), 148.0 (C-6), 141.9 (C-9), 137.7 (C-4'), 133.1 (C-1'), 128.6 (C-10), 109.6 (C-8), 108.1 (C-2'), 108.1 (C-6'), 105.0 (C-5), 102.7 (-OCH_2_O-), 66.6 (C-3a ), 59.6 (4'-OMe), 55.5 (3', 5'-OMe), 45.8 (C-2), 44.6 (C-1), 43.4 (C-3). As analyzed above, it was identified as podophyllotoxone [34,35].

Compound 13, colorless crystal; m.p. 200~202°C; molecular formula: C_8_H_8_O_4_; ^1^H NMR (CD_3_COCD_3_, 500 MHz) *δ*: 7.60 (1H, dd, J = 8.0 Hz, J = 1.7 Hz, H-6), 7.57 (1H, d, J = 1.7 Hz, H-2), 6.92 (1H, d, J = 8.0 Hz, H-5), 3.89 (3H, s, OCH_3_); ^13^C NMR(CD_3_COCD_3_, 125 MHz) *δ*: 166.7 (C = O), 152.0 (C-3), 147.2 (C-4), 124.0 (C-6), 122.0 (C-1), 114.6 (C-5), 112.6 (C-2), 55.4 (-OCH_3_). As analyzed above, it was identified as vanillic acid [36].

Compound 14, colorless power; m.p. 238~240°C; molecular formula: C_21_H_20_O_7_; ^1^H NMR (CD_3_COCD_3_, 500MHz) *δ *: 6.75 (1H, s, H-5), 6.52 (1H, s, H-8), 6.39 (2H, s, H-2', H-6'), 5.96 (2H, d, *J *= 2.9 Hz, OCH_2_O), 4.55 (1H, d, *J *= 5.2 Hz, H-1), 4.43 (1H, dd, *J *= 8 Hz, *J *= 6.9 Hz, 3a-*α*H ), 3.97 (1H, dd, *J *= 10.3 Hz, *J *= 8 Hz, 3a-*β*H ), 3.69(6H, s, 3'-OM e, 5'-OM e), 3.12~3.09 (1H, m, 4-*α*H), 2.86~2.76 (3H, m, 4-*β*H, H-2, H-3); ^13^C NMR (CD_3_COCD_3_, 125 MHz) *δ*: 174.8 (C-2a), 147.1 (C-3'), 147.1 (C-5'), 147.0 (C-6), 146.6 (C-7), 131.8 (C-1'), 131.8 (C-4'), 131.4 (C-9), 129.3 (C-10), 110.1 (C-8), 108.8 (C-5), 108.4 (C-2'), 108.2 (C-6'), 101.2 (-OCH_2_O-), 71.6 (C-3a), 55.8 (3'-OMe, 5'-OMe), 46.9 (C-2), 43.6 (C-1), 32.9 (C-4), 32.6(C-3). As analyzed above, it was identified as 4'-demethyldeoxypodophyllotoxin [37].

Compound 15, colorless cubic crystal; m.p. 164~166°C; molecular formula: C_12_H_22_O_11_; ^1^H NMR (D_2_O, 500 MHz) *δ*: 5.37 (1H, d, *J *= 4 Hz, H-1), 4.17 (1H, d, *J *= 8.6 Hz, H-3'), 4.00 (1H, t, *J *= 8.6 Hz, H-5'), 3.83~3.76 (1H, m, H-4'), 3.78~3.71 (5H, m, H-6, 6', 4'), 3.70 (1H, dd, *J *= 9.5 Hz, H-5 ), 3.63(2H, s, H-1'), 3.50 (1H, dd, *J *= 3.8 Hz, H-3), 3.41 (1H, t, *J *= 10 Hz, H-4); ^13^C NMR (D_2_O, 125 MHz) *δ*: 103.7 (C-2'), 92.2 (C-1), 81.4 (C-3'), 76.3 (C-4'), 73.9 (C-5'), 72.5 (C-5), 72.4 (C-3), 71.1(C-2), 69.2 (C-4), 62.3 (C-1'), 61.0(C-6'), 60.3 (C-6). As analyzed above, it was identified as sucrose [38].

### Anticancer activity

**Figure 2:**
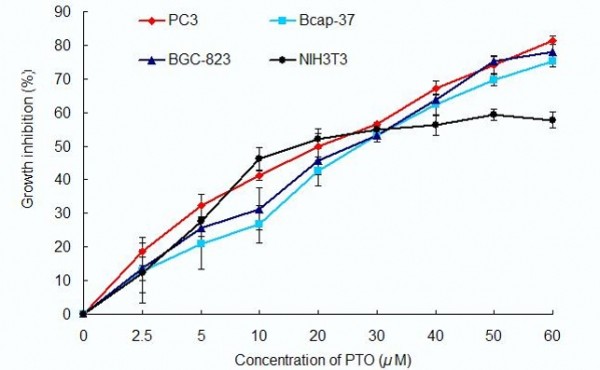
**Effect of PTO on proliferation of tumor cells**. After PC3, Bcap-37, BGC-823 and NIT3T3 cells were treated with PTO for 72 h in the concentrations varied from 2.5 to 60 *μ*M, growth inhibition of those tumor cells was detected by MTT assay. Data are presented as means ± SD, n = 4.

**Figure 3:**
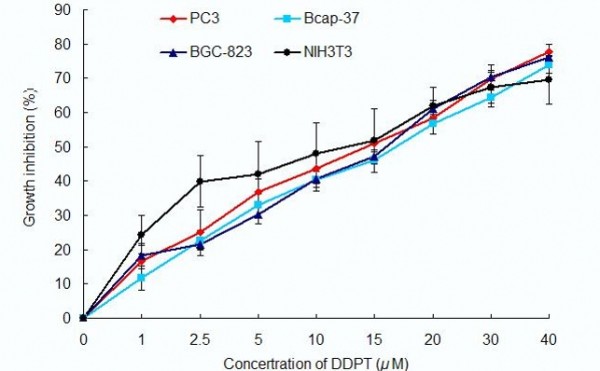
**Effect of DDPT on proliferation of tumor cells**. After PC3, Bcap-37, BGC-823 and NIT3T3 cells were treated with DDPT for 72 h in the concentration varied from 1 to 40 *μ*M, growth inhibition of those tumor cells was detected by MTT assay. Data are presented as means ± SD, n = 4.

The potential effect of the extracts from *D. versipellis *was investigated on the viability of PC3, Bcap-37, BGC-823 and NIH3T3 cells using MTT assay at the concentration of 20 *μ*M, with ADM (adriamycin) [39] being used as the positive control [40,41]. MTT [3-(4, 5-dimethylthiazol-2-yl)-2, 5-diphenyltetrazolium bromide] assay is a common method of measuring the proliferation of cells. The results are summarized in Table 1. It could be seen that PTO and DDPT possess potent activities against the three human cancer cell lines tested. The inhibitory ratios of PTO and DDPT at 72 h after treatment were 52.0% and 67.1% against PC3 cells, 42.1% and 56.6% on Bcap-37 cells, 47.9% and 60.7% on BGC-823 cells, and 43.7% and 59.8% on NIH 3T3 cells. Further experiments found that proliferation of these three carcinoma cells were significantly inhibited by PTO and DDPT in a concentration-dependent manner, as shown in Figures 2 and 3. The IC_50 _values of PTO on PC3, Bcap-37 and BGC-823 cells were (17.8±1.0) *μ*M, (21.1±1.8) *μ*M, (19±1.6) *μ*M respectively, while for DDPT, the IC_50 _values were (10.6±1.5) *μ*M, (13.2±0.5) *μ*M, (11.5±0.6) *μ*M, respectively, which were both lower than that on NIH 3T3 cells [(24.2±2.1) *μ*M for PTO; (16.2±9.9) *μ*M for DDPT]. The results showed that PTO and DDPT had more potent activities against PC3, Bcap-37 and BGC-823 cells than on NIH3T3 cells. Besides, the inhibitory effect on tumor cells of DDPT was stronger than that of PTO.

**Table 1:** Growth inhibition effect of various constituents of *D. versipellis *on different cells

Compound (20 *μ*M)	Growth inhibition (%)			
	
	PC3	Bcap-37	BGC-823	NIT3T3
*β*-sitosterol	14.7 ± 8.5	10.9 ± 10.4	19.5 ± 9.3	1.7 ± 9.3
4'-demethylpodophyllotoxin	60.5 ± 5.2**	56.7 ± 9.1**	56.9 ± 12.4	60.1 ± 6.1**
Kaempferol	43.9 ± 11.9**	53.2 ± 10.9**	53.4 ± 14.9	39.2 ± 8.2**
picropodophyllotoxin-4-*O*-glucoside	9.0 ± 9.9	7.4 ± 9.3	24.6 ± 11.3**	6.4 ± 8.8
kaempferol-3*-O-β-D*-glucopyranoside	23.0 ± 6.4**	29.9 ± 10.8**	13.2 ± 8.7	23.0 ± 7.9**
4'-demethylpodophyllotoxin-4-*O*-glucoside	19.8 ± 7.1**	21.9 ± 6.5**	14.5 ± 8.0	6.5 ± 7.7
quercetin-3-*O-β-D*-glucopyranoside	11.6 ± 9.1	15.2 ± 10.1	40.7 ± 11.9**	5.1 ± 9.9
icropodophyllotoxin-4-O-β-D-glucopyranosyl-(1→6)-*β-D*-glucopyranoside.	10.2 ± 8.1	23.9 ± 10.2	23.6 ± 10.7**	2.2 ± 8.8
Quercetin	29.8 ± 8.3	39.0 ± 13.3**	22.1 ± 4.3*	8.1 ± 5.0
Podophyllotoxone	52.0 ± 5.6**	42.1 ± 6.3**	47.9 ± 8.1	43.7 ± 6.2**
vanillic acid	29.2 ± 3.9**	30.3 ± 10.9**	28.5 ± 12.3	10.2 ± 5.0*
4'-demethyldeoxypodophyllotoxin	67.1 ± 3.4**	56.6 ± 12.3**	60.7 ± 9.8**	59.8 ± 3.5**
ADM	93.2 ± 1.6	90.6 ± 1.2**	98.3 ± 4.0**	97.5 ± 1.7**

All the values are expressed as mean ± SD (n = 6). * P < 0.05, ** P < 0.01 compared with control.

To determine whether the growth inhibitory activity of PTO and DDPT were related to the induction of apoptosis, the morphological character changes of PC3 and Bcap-37 cells were investigated using the AO/EB staining and Hoechst 33258 staining under ﬂuorescence microscopy.

**Figure 4:**
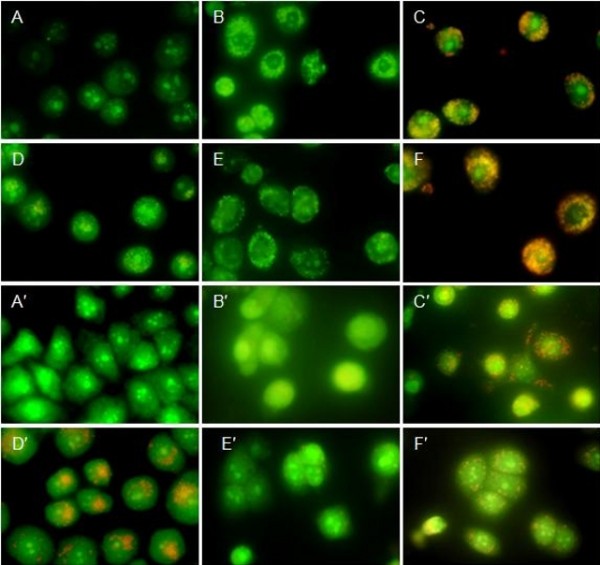
**Nuclei morphological changes during PTO and DDPT-induced apoptosis in tumor cells detected by AO/EB staining**. After treated with PTO and DDPT at 20 *μ*M, PC3 and Bcap-37 cells were stained with AO/EB (100 *μ*g/mL) and observed under ﬂuorescence microscopy. For PC3 cells group, A: negative control (without treatment); D: positive control, treated with HCPT (20 *μ*M) for 24 h; B and C: treated with PTO (20 *μ*M) for 24, 48 h; E and F, treated with DDPT (20 *μ*M) for 24, 48 h, respectively. For Bcap-37 cells group, A': negative control; D': positive control, treated by HCPT (20 *μ*M) for 24 h; B' and C': treated with PTO (20 *μ*M) for 24, 48 h; E'and F': treated with DDPT (20 *μ*M) for 24, 48 h, respectively.

Since acridine orange (AO) was a vital dye that could stain nuclear DNA across an intact cell membrane and ethidium bromide (EB) could only stain cells that had lost membrane integrity. Thus, live cells will be uniformly stained green, early apoptotic cells will be densely stained as green yellow or displayed green yellow fragments, while late apoptotic cells will be densely stained as orange or displayed orange fragments, and necrotic cells will be stained with orange with no condensed chromatin could be found by the AO/EB doubly staining. After tumor cells were treated with PTO and DDPT (20 *μ*M) for 24, 48 h, the morphological changes were analyzed. As shown in Figure 4, green live PC3 and Bcap-37 cells with normal morphology were seen in the negative control group (Figure 4A and 4A'). In contrast, early apoptotic cells with yellow green dots in PC3 cell nuclei and late apoptotic cells with orange dots in Bcap-37 cell nuclei could be seen in the positive control group (Figure 4D and 4D'), meanwhile bright green early apoptotic cells with nuclear margination and chromatin condensation occurred in the experimental group with 24 h of treatment (Figure 4B and 4E) and orange colored apoptotic cells with apoptotic bodies and chromatin fragmentation could be seen when PTO and DDPT were applied for 48 h (Figure 4E and 4F). Similarly, morphological changes of Bcap-37 cell apoptosis were also observed under microscope (Figure 4B', 4C', 4E', and 4F'). The results suggested that PTO and DDPT were able to induce apoptosis in PC3 and Bcap-37 cells.

**Figure 5:**
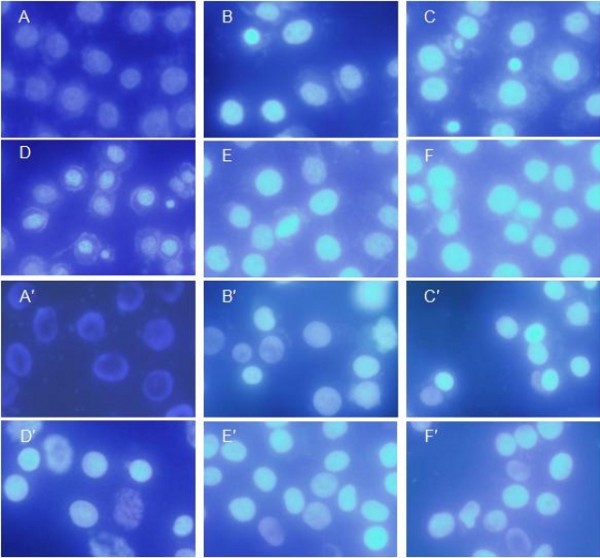
**Nuclei morphological changes during PTO and DDPT-induced apoptosis in tumor cells detected by Hoechst 33258 staining**. Tumor cells treated with PTO and DDPT (20 *μ*M) were stained by Hoechst 33258 and observed under ﬂuorescence microscopy. For PC3 cells group, A: negative control (without treatment); D: positive control, treated with HCPT (20 *μ*M) for 24 h; B and C: treated with PTO (20 *μ*M) for 24, 48 h; E and F: treated with DDPT (20 *μ*M) for 24, 48 h, respectively. For Bcap-37 cells group, A': negative control (without treatment); D': positive control, treated with HCPT (20 *μ*M) for 24 h; B' and C': treated with PTO (20 *μ*M) for 24, 48 h; E' and F': treated with DDPT (20 *μ*M) for 24, 48 h, respectively.

Hoechst 33258 staining was also carried out to investigate the apoptosis induction of PTO and DDPT (20 *μ*M) on PC3 and Bcap-37 cells. Membrane-permeable Hoechst 33258 was a blue fluorescent dye and stained the cell nucleus. When cells were treated with Hoechst 33258, live cells with uniformly light blue nuclei were observed under fluorescence microscope, while apoptotic cells exhibited bright blue because of karyopyknosis and chromatin condensation, and the nuclei of dead cells could not be stained. The experimental results were shown in Figure 5. Compared with the negative control (Figure 5A and 5A'), a part of cells with smaller nuclei and condensed staining appeared in the positive control group (Figure 5D and 5D'). After treated with PTO and DDPT for a given time, some PC3 cell nuclei became pyknotic (shrunken and dark), as shown in Figure 5B, 5C, 5E and 5F. Besides, Bcap-37 cells treated with PTO and DDPT for 24 h had no obvious morphologic changes (Figure 5B' and 5E'), but most cell nuclei appeared to be highly condensed (brightly stained) after 48 h of treatment (Figure 5C' and F'). The results once again indicated that PTO and DDPT could induce apoptosis in PC3 and Bcap-37 cells.

TUNEL assay was further carried out to confirm the cell apoptosis inducing activities of PTO and DDPT.

**Figure 6:**
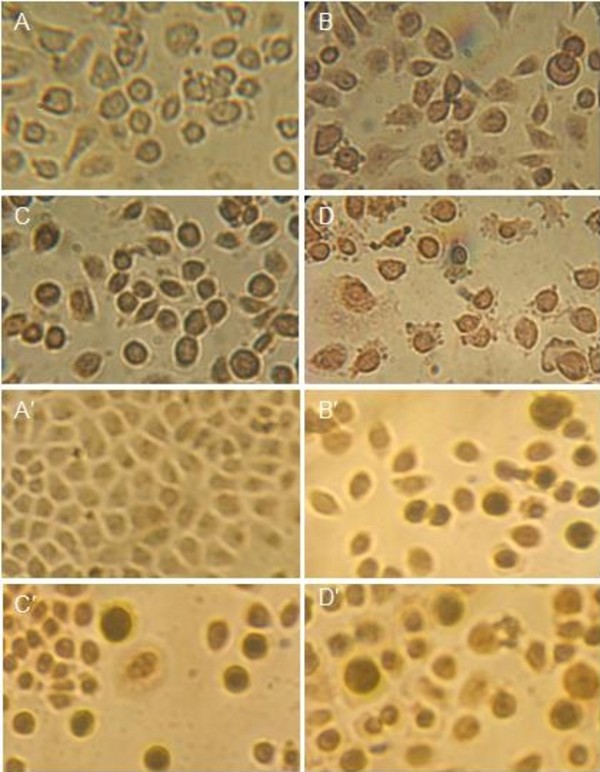
**Nuclei morphological changes during PTO and DDPT-induced apoptosis in PC3 and Bcap-37 cells detected by TUNEL assay**. Tumor cells treated with PTO and DDPT (20 *μ*M) were assayed by TUNEL and observed under light microscopy. For PC3 cells group, A: negative control (without treatment); C: positive control, treated with HCPT (20 *μ*M) for 24 h; B and D: treated with PTO and DDPT (20 *μ*M) for 24 h; For Bcap-37 cells group, A': negative control (without treatment); C': positive control, treated with HCPT (20 *μ*M) for 24 h; B' and D': treated with PTO and DDPT (20 *μ*M) for 24 h.

TUNEL (Terminal deoxynucleotidyl Transferase Biotin-dUTP Nick End Labeling) is a popular method for identifying apoptotic cells *in situ *by detecting DNA fragmentation. Due to degradation of DNA that resulted from the activation of Ca/Mg dependent endonucleases in apoptotic cells, DNA cleavage occurred and led to breaking of strand within the DNA. These strand breaks of cleaved DNA could be identified by terminal deoxynucleotidyl transferase (TdT) that catalyzed the addition of biotin-dUTP. The biotin-labeled cleavage sites were then detected by reaction with streptavidin-HRP and visualized by DAB indicating a brown color. As shown in Figure 6, most nuclei were stained as a discernible brown in the treatment groups with HCPT (Figure 6C and 6C'), PTO (Figure 6B and 6B'), and DDPT (Figure 6D and 6D') compared with the control (Figure 6A and 6A').

**Figure 7:**
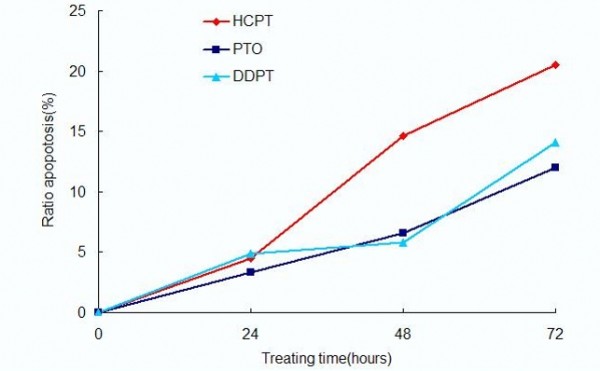
**The apoptosis ratios of PC3 cells treated with PTO and DDPT (20 *μ*M) assessed by ﬂow cytometry**. These cells were treated with HCPT, PTO and DDPT (20 *μ*M) for 24, 48 and 72 h.

**Figure 8:**
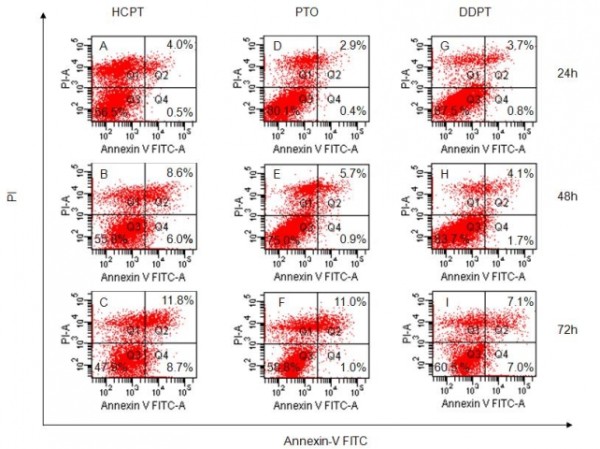
**Flow cytometry analysis for apoptosis inducing activities of PTO and DDPT on PC3 cells**. The appearance of apoptosis cells was detected by flow cytometry using Annexin V/PI staining. In the figure, A, B and C: treated with HCPT (20 *μ*M) for 24, 48 and72 h; D, E and F: treated with PTO (20 *μ*M) for 24, 48 and 72 h; G, H and I: treated with DDPT (20 *μ*M) for 24, 48 and 72 h.

In addition, the apoptosis ratios induced by PTO and DDPT caused apoptosis in tumor cells was quantitatively assessed by flow cytometry. In the early stages of apoptosis, phosphatidylserine (PS) was translocated from the inside of the cell membrane to the outside. Annexin V, a calcium dependent phospholipid-binding protein associated with a high affinity for phosphatidylserine, was used to detect early apoptotic cells. PI (Propidine Iodide) was a red fluorescent dye and stained cells that had lost membrane integrity. So, cells stained with FITC-annexin V and PI were discriminated necrotic cells (Q1, Annexin^-^/PI^+^), late apoptotic cells (Q2, Annexin^+^/PI^+^), intact cells (Q3, Annexin^-^/PI^-^) and early apoptotic cells (Q4, Annexin^+^/PI^-^). As shown in Figure 7, PTO and DDPT (20 *μ*M) could induce apoptosis of PC3 cells, and highest apoptosis ratios, 12.0% and 14.1% for PTO and DDPT respectively, were obtained after 72 h of treatment at a concentration of 20 *μ*M. Furthermore, as shown in Figure 8, the early (Q4) and late (Q2) apoptosis of PC3 cells which treated with PTO and DDPT increased gradually in a time-dependent manner. The late apoptotic ratio of cells increased to approximately 11.0% at 72 h after treatment of PTO (20 *μ*M), which was close to that of positive control HCPT (11.8%). And the highest rate of early apoptosis was 7.0% when cells were treated with DDPT at the concentration of 20 *μ*M for 72 h.

In summary, these results indicated that inhibitive effects observed in response to PTO and DDPT were associated with induction of apoptotic cell death.

## Conclusions

*Dysosma versipellis *(Hance) M. Cheng, an important medicinal plant species, is considered 'endangered' by the China Species Red List and has been considered as vulnerable by the IUCN due to its rapid decline [42,43]. Therefore, studies on the chemical constituents from *D. versipellis *and their biological activities have assumed significance for the rational development and utilization of this plant. In our study, fifteen compounds were extracted and identified from *D. versipellis *grown in Guizhou province, and the cell growth inhibition effects of these constituents on PC3, Bcap-37, and BGC-823 cells were carried out by MTT assay. Among these compounds, PTO, which was extracted from *D. versipellis *for the first time, together with DDPT showed potent activities on PC3, Bcap-37, and BGC-823 cells in a dose-dependent manner. And the IC_50 _values of PTO and DDPT on three cell lines were (17.8±1.0) *μ*M, (21.1±1.8) *μ*M, (19 ±1.6) *μ*M and (10.6±1.5) *μ*M, (13.2±0.5) *μ*M, (11.5±0.6) *μ*M, respectively.

The apoptosis inducing activities of PTO and DDPT on PC3 and Bcap-37 cells were investigated through AO/EB staining, Hoechst 33258 staining, TUNEL and ﬂow cytometry analysis assay. The results demonstrated that PTO and DDPT from *D. versipellis *have potential to be employed in adjuvant therapy for treating human prostate and breast tumors. Further studies of the specific mechanisms of these compounds on human malignant tumors are currently underway.

## Competing interests

The authors declare that they have no competing interests.

## Authors' contributions

XQX, XHG performed the experiments, analyzed the data and wrote the paper. KY performed the experiments, LHJ, DH planned and analyzed the data and BAS, SY planned the experiments, wrote the paper and give final approval of the version to be published. All authors contributed to this study, read and approved the final manuscript.
